# Pylephlebitis Associated with Inferior Mesenteric Vein Thrombosis Treated Successfully with Anticoagulation and Antibiotics in a 37-Year-Old Male

**DOI:** 10.1155/2020/3918080

**Published:** 2020-02-13

**Authors:** Mohamed Abdallah, Ahmad Gohar, Smitha Naryana Gowda, Hafez M. Abdullah, Ali Al-hajjaj

**Affiliations:** Internal Medicine, University of South Dakota Sanford School of Medicine, Vermillion, SD 57069, USA

## Abstract

Pylephlebitis is a condition in which there is septic thrombophlebitis of the portal-mesenteric venous system. It is a rare condition that usually arises as a complication of an intra-abdominal infection or inflammation. Being rare, it may be overlooked as a differential in cases of abdominal pain when the more common causes have been excluded. We present a case of a 37-year-old Hispanic male who presented with acute abdominal pain and loose stools. He was initially treated for acute gastroenteritis but his clinical condition deteriorated. He was eventually diagnosed with pylephlebitis with associated inferior mesenteric vein thrombosis. He was successfully treated with IV antibiotics and warfarin. Pylephlebitis should not be overlooked as a differential in an appropriate clinic setting as it has a high mortality rate.

## 1. Introduction

Pylephlebitis or portal vein septic thrombophlebitis, defined as suppurative thrombophlebitis of the portal-mesenteric venous system, is a very rare condition. It complicates less than 0.2% of all intra-abdominal infections with an estimated annual incidence of 0.37–2.7 cases per 100,000 inhabitants per year [1–3]. Inferior mesenteric vein thrombosis complicating pylephlebitis is exceedingly uncommon, complicating less than 2% of pylephlebitis cases [4]. Despite its rarity, portal vein thrombosis in the setting of intra-abdominal infection carries a high mortality, estimated to be more than 20% [1, 4]. Here, we report a rare case of pylephlebitis with associated thrombosis involving inferior mesenteric vein. The patient was managed with prolonged IV antibiotics and anticoagulation and made a successful recovery.

## 2. Case Presentation

A 37-year-old male presented to the emergency department with fever, vomiting, watery diarrhea, and diffuse abdominal pain for three days. His past medical history was significant for migraine headaches and alcohol abuse. He did not have a history of cirrhosis. He denied any blood in stools, rashes, neck stiffness, or recent travel. He worked as a construction worker and denied any sick contacts.

Physical examination was pertinent for tachycardia with a heart rate of 147 beats per minute, respiratory rate of 25 breaths per minute, temperature of 99.5°F, and oxygen saturation of 100% on room air. Blood pressure was 101/59 mmHg. The rest of his physical examination was unremarkable.

On laboratory workup, his hemoglobin was 15.3 g/dL (13.5–17.5 g/dL), white blood cells count was 2,900 cells/uL(4.5–11 K/uL), total bilirubin was 2.7 mg/dL (0.3–1.0 mg/dL), aspartate aminotransferase 152 U/L (13–39 U/L), alanine aminotransferase 167 U/L (4–33 U/L), alkaline phosphatase 174 U/L (34–104 U/L), lipase 68 U/L (11–82 U/L), prothrombin time 14.3 sec (11.8–14.9 sec), and aPTT 32.9 (24–36 sec).

**Figure 1:**
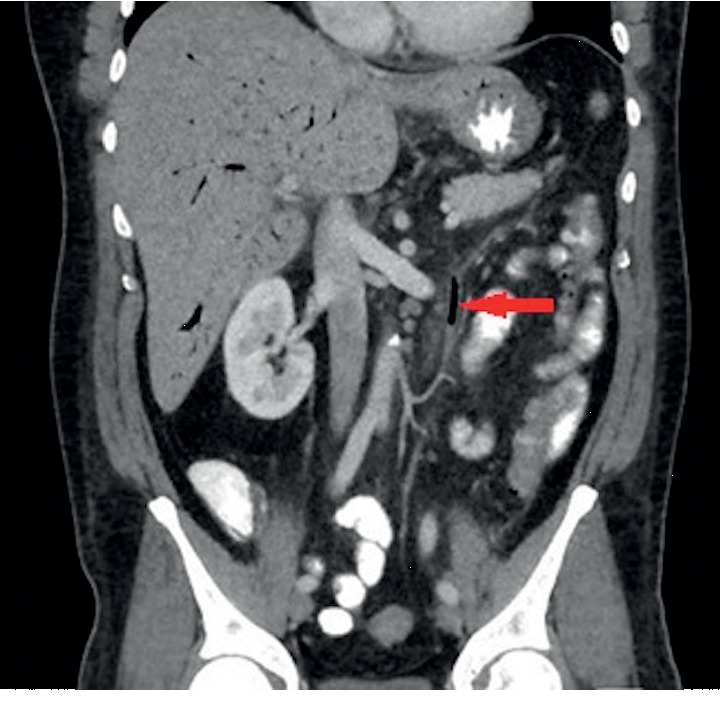
Computed tomography (CT) of the abdomen and pelvis with intravenous contrast showed gas in the inferior mesenteric vein.

**Figure 2:**
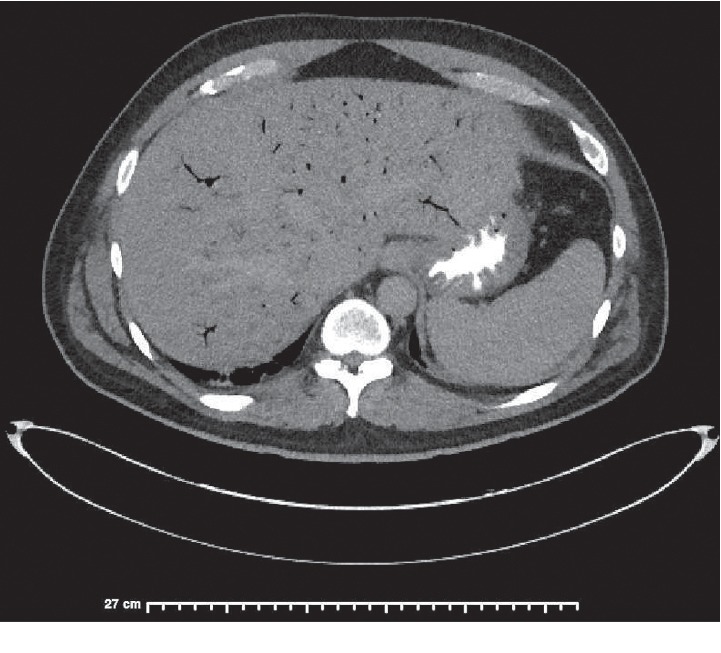
Computed tomography (CT) of the abdomen and pelvis with intravenous contrast showed a large amount of portal venous gas.

A preliminary diagnosis of sepsis due to viral or bacterial gastroenteritis was made, and supportive management with intravenous (IV) fluids and antipyretics was initiated. However, his clinical condition deteriorated. On hospital day 5, he developed a fever of 102.5°F, chills and hypotension. White cell count rose to 17,200 cells/uL (4.5–11k cells/uL). Chest-X-ray, urinalysis, and abdominal ultrasound were unremarkable. An Ultrasound and MRCP only showed a small amount of fluid around the gallbladder. A HIDA scan was undertaken, which ruled out cholecystitis as the cause of the elevated liver enzymes and sepsis. Computed tomography (CT) of the abdomen and pelvis with intravenous contrast showed thickening and pericolonic stranding of descending colon and sigmoid colon, a large amount of portal venous gas, gas in the inferior mesenteric vein, and low attenuation filling defects of the inferior mesenteric vein (IMV) suggestive of septic thrombosis (Figures 1 and 2).

Meropenem 2 g every 8 hours was started for a presumable intra-abdominal bacterial infection. The patient's clinical condition improved over the following 48 hours. Blood cultures grew Bacteroides fragilis, Clostridium fragilis, and Fusobacterium necrophorum.

Given presence of concomitant bacteremia and inferior mesenteric vein thrombosis in light of coexisting colitis/diverticulitis, a diagnosis of pylephlebitis was made, and the patient was started on unfractionated heparin infusion in addition to the meropenem therapy.

**Figure 3:**
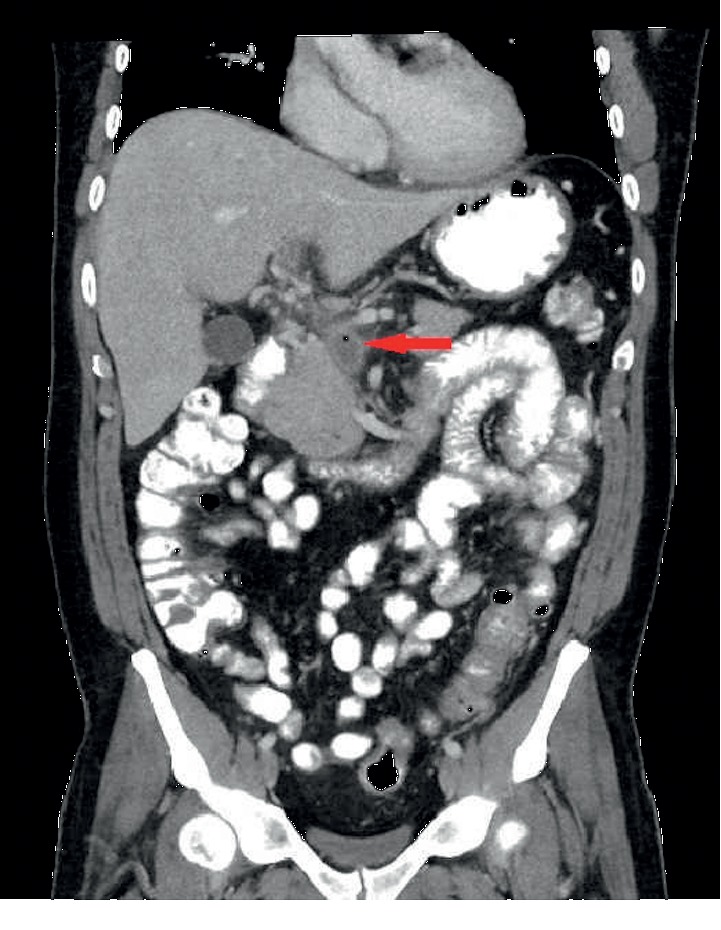
Computed tomography (CT) abdomen was repeated which showed resolution of the portal vein gas but showed interval development of portal vein thrombosis and proximal superior mesenteric vein.

The patients' clinical status improved over course of the following week with improvement of the abdominal pain and resolution of fever. The patient's transaminases started to trend down, and the patient was discharged home with IV ertapenem for one month and oral warfarin for anticoagulation. The liver enzymes normalized as the patient was followed up in the outpatient clinic. His abdominal symptoms resolved but his ESR remained elevated at 115 mm/hour, so CT abdomen was repeated which showed resolution of the portal vein gas but showed interval development of portal vein thrombosis and proximal superior mesenteric vein (Figure 3). Despite this radiologic progression, the patient's clinical status was improving as mentioned earlier. The case was discussed in a multidisciplinary team meeting involving hepatology, gastroenterology, hematology, and infectious disease teams. A decision was made to continue anticoagulation with Coumadin for a total 6 months duration and to do colonoscopy to evaluate for underlying inflammatory or neoplastic process beside the infectious process. Colonoscopy revealed sigmoid diverticulosis supporting diverticulitis as the etiology of pylephlebitis. The patient continued to follow-up in the clinic and has remained asymptomatic since then.

## 3. Discussion

Pylephlebitis, first described in 1846 by Waller, is an uncommon yet serious condition that can complicate intra-abdominal infections [5]. A review of more than 100 published case reports of pylephlebitis from 1971 to 2009 showed that the mean age of presentation is 42.3 years. Acute pylephlebitis, which is diagnosed if symptoms start within 60 days of presentation, was seen in 81% of patients. Regarding clinical features, fever, abdominal pain, hepatomegaly, splenomegaly, transient, or permanent ascites are observed in 86%, 82%, 42%, 23%, and 21% of patients, respectively [4]. Leukocytosis is observed in 80% of patients, elevation of AST and/or ALT in 69%, alkaline phosphatase (ALP) and/or gamma-glutamyl transpeptidase (GGT) levels in 40%, total bilirubin in 55%, and anemia in 55% [4].

Diverticulitis is the most common etiology leading to pylephlebitis and is seen in 19–30% of patients, followed by pancreatitis (5–30%) and appendicitis (19%) [1, 4]. Associated conditions were recent abdominal surgery (19%), remote abdominal surgery in (18%), and immunosuppression (14%). Cultures from blood or other tissue were positive in 44–77% [1, 4]. In half of the patients, a polymicrobial infection is observed [4]. The most commonly isolated organisms are Bacteroides fragilis (27%), followed by Escherichia coli (22%) and Streptococcus spp (17%) [1, 4].

Diagnosis can be established through abdominal imaging using computerized tomography (CT), ultrasonography, or magnetic resonance imaging (MRI). However, the diagnostic modality of choice is intravenous contrast-enhanced CT, which can demonstrate thrombi (hypodense filling defects) and/or gas in the portal system (seen in 18% of cases) [6]. Unlike pneumobilia (the presence of gas in the biliary system), gas in the portal venous system extends to the hepatic periphery allowing differentiation between the two entities on imaging studies [6–8]. Complications of pylephlebitis include extension to the superior mesenteric vein (SMV) in 42%, thrombosis of the intrahepatic branches of PV (39%), liver abscesses (37%), splenic vein (12%), and inferior mesenteric vein thrombosis (2%). Of note, intestinal ischemia complicates up to 25% of patients with SMV thrombosis [4].

Management with antibiotics therapy is indicated in all patients. Anticoagulation therapy is controversial but has shown favorable outcomes, with more complete recanalization decreased mortality, compared to antibiotics therapy alone. Anticoagulation should be strongly considered in case of extension of the thrombosis to the mesenteric system, lack of response to monotherapy with antibiotics, persistence of fever, and the existence of thrombophilic condition [4, 9]. However, a case-by-case evaluation is recommended before offering anticoagulation [4]. Increased morbidity and mortality is observed in patients with significant comorbidities (liver transplantation, CRF, DM, burns, malignancies, and IBD), with sepsis and peritonitis being the most frequent causes of mortality [4].

## 4. Conclusions

Pylephlebitis is a condition in which there is septic thrombophlebitis of the portal-mesenteric venous system. It is a rare condition that usually arises as a complication of an intra-abdominal infection or inflammation. Being rare, it may be overlooked as a differential in cases of abdominal pain when more common causes have been excluded. It is treated with IV antibiotics and anticoagulation. Pylephlebitis should not be overlooked as a differential in an appropriate clinic setting as it has a high mortality rate.
